# Analysis of team success based on match technical and running performance in a professional soccer league

**DOI:** 10.1186/s13102-022-00473-7

**Published:** 2022-05-05

**Authors:** Marcin Andrzejewski, José M. Oliva-Lozano, Paweł Chmura, Jan Chmura, Sławomir Czarniecki, Edward Kowalczuk, Andrzej Rokita, José M. Muyor, Marek Konefał

**Affiliations:** 1 Department of Methodology of Recreation, Poznań University of Physical Education, Królowej Jadwigi 27/39, 61871 Poznań, Poland; 2 Health Research Centre, University of Almería, Ctra. Sacramento s/n. 04120, La Cañada de San Urbano, Almería, Spain; 3 Department of Team Games, Wrocław University of Health and Sport Sciences, I.J. Paderewskiego 35, 51612 Wrocław, Poland; 4 Department of Human Motor Skills, Wrocław University of Health and Sport Sciences, I.J. Paderewskiego 35, 51612 Wrocław, Poland; 5 Football Club, Bayer 04 Leverkusen, Bismarckstr. 122 – 124, 51373 Leverkusen, Germany; 6 Football Club, Hannover 96, Robert-Enke-Str. 1, 30169 Hannover, Germany; 7 Laboratory of Kinesiology, Biomechanics and Ergonomics (KIBIOMER Lab). Research Central Services, University of Almería, Almería, Spain

**Keywords:** Football, Match analysis, Tracking, Load, Physical and technical activity

## Abstract

**Background:**

The aims of this study were to (1) determine the match technical and running performance required by different teams based on their final ranking position in a professional soccer league; and (2) analyze the correlation between teams’ success at the end of the season and variables related to match technical and running performance.

**Methods:**

These performance data were collected during a total of 612 matches in the German Bundesliga. The final ranking position and the total of points obtained by each team at the end of the season were registered for the analysis of the correlation between team success and performance.

**Results:**

The main findings were that there was a significant interaction (*p* < 0.05) between the ranking position, and both match technical and running performance. However, goals scored, saved shots on goal by the goalkeeper, assists, allowed shots on goal, goals conceded, ball possession ratio and successful passes from open play were the variables with the strongest correlation (*r* > 0.7; *p* ≤ 0.01) with the total of points obtained at the end of the season.

**Conclusions:**

Strength and conditioning coaches may consider these results to develop adequate training strategies, which may not only optimize performance but also reduce the injury risk.

## Background

Time-motion analysis technologies have assisted sport scientists and coaches to gain a thorough understanding of the physical requirements of soccer [1]. The development of time-motion analysis technologies has allowed a better description of the movement profile since movement patterns can be reported in a quantifiable manner [2]. Then, training strategies can be carefully implemented and manipulated to prepare the players for the requirements of the competition. Currently, the external load of a team sport athlete can be measured by these tracking technologies, which include global positioning systems, local positioning systems, and optical tracking systems [1]. Furthermore, the semi-automated systems track players’ movement through a camera-based system, which allow the collection of both physical and technical activity.

Soccer players are continually engaged in multidirectional physical and technical activities [3, 4]. Specifically, previous studies have showed that the team success at the end of the season was associated with the technical performance [5–9]. For example, a recent study in the German Bundesliga showed that there seems to be a tendency that accuracy-related variables (e.g., shots on target or goal efficiency) are critical [5, 10]. However, another study in the Chinese Super League concluded that there were other indicators such as ball possession or won duels which were associated with team success at the end of the season [9]. From a practical perspective, this implies that match technical performance indicators could be used for talent identification and training purposes while considering the evolution of technical performance soccer in professional soccer [11, 12].

In addition, physical performance may play an auxiliary role to successful technical performance [9, 13–15]. For example, a previous study concluded that match running performance alone was not significantly correlated with team success, but positive-significant correlations were found for running performance with ball possession [13]. In addition, a recent study observed that low-ranked teams covered greater distance covered without ball possession than higher-ranked teams [9]. Based on an investigation in the Spanish LaLiga, soccer is evolving to a more intense game in which players have increased the total of sprints and high-intensity distances covered in match [16]. However, there are variables related to match running performance such as players’ maximum running speed, which had a poor correlation with team success according to a recent study [17].

In this regard, research regarding the correlation between team success, match technical and running performance in professional soccer is necessary because little data are available, especially in the German Bundesliga. In addition, previous studies have focused on the relationship between team success and technical performance [5] or between team success and match running performance [18]. However, the integration of both match technical and running performance variables in the same analysis is necessary to have a better understanding of team success. Knowledge about match technical and running performance allows practitioners to manage the training load, which is key to improve performance and reduce the injury risk [16]. Therefore, the aims of this study were to: (1) determine the match technical and running performance required by high-ranked, medium-ranked, and low-ranked teams in a professional soccer league; and (2) analyze the correlation between teams’ success at the end of the season and variables related to match technical and running performance.

## Methods

### Study design

Match technical and running performance data, which were collected by electronic performance and tracking systems during 612 matches in two consecutive seasons (2017–2018 and 2018–2019), were analyzed. Also, the total of points obtained at the end of each season were registered in order to analyze the correlation between the team success and the variables related to match technical and running performance.

### Participants

Data were collected from a total of 1224 match observations, which included professional soccer players (*n* = 461) of the German Bundesliga. However, goalkeepers were not included in the study considering the different activity-profile of this playing position [19]. In addition, anonymity of the players was maintained, and the study was approved by an Institutional Ethics Committee (no. 12/2021). Restrictions apply to the availability of the data analyzed during the current study considering that it is based on data collected in the context of professional soccer matches and allowed all clubs of the league to match data analysis, and no informed consent was required by the Ethics Committee.

### Procedures

The kinematic data analysis was conducted through IMPIRE AG (Ismaning, Germany) match analysis system, which was at a sampling frequency of 25 Hz. IMPIRE AG (Ismaning, Germany) and Cairos Technologies AG (Karlsbad, Germany) provide a vision-based tracking system called VIS.TRACK. Specifically, the players movements were recorded by two cameras [20] and the system used state-of-the-art algorithms as well as 2D and 3D video-recording technology for a detailed kinematic analysis of each soccer match.

Once the seasons were over, both match technical and running performance variables were downloaded. The technical performance variables included: goals scored, goals conceded, shots on goal, shots on goal allowed, missed shots on goal, shots off target, blocked shots on goal by any player, saved shots on goal by the goalkeeper, successful passes from open play, unsuccessful passes from open play, successful crosses from open play, unsuccessful crosses from open play, successful passes from set pieces, unsuccessful passes from set pieces, successful crosses from set pieces, unsuccessful crosses from set pieces, clearances, corner kicks, assists, aerial duels won, aerial duels lost, ground duels won, ground duels lost, counter attacks, offsides, fouls generated by the team, fouls generated by the opponent team, hand games, yellow cards, yellow–red cards (i.e., second yellow card in the match), red cards, and percentage of ball possession. In addition, match running performance variables included maximum speed reached in the match, total running distance (TD) covered with and without ball possession, sprinting distance (SPD, above 22.68 km/h) covered with and without ball possession as well as the total of sprinting actions (SPA, above 22.68 km/h) with and without ball possession. The speed thresholds for sprinting actions were set based on previous studies [13, 21].

Moreover, the final ranking position at the end of each season was used to categorize the teams into high-ranked teams (from the first to the sixth team), medium-ranked teams (from the seventh to the fifteenth team), and low-ranked teams (from the sixteenth to the eighteenth team) according to previous research [18, 22]. Also, the total of points achieved by the teams at the end of each season were registered in order to analyze the correlation between the team success and match technical and running performance.

### Statistical analysis

First of all, descriptive statistics of the match running demands and technical performance required by high-ranked, medium-ranked, and low-ranked teams were calculated. Also, a multivariate analysis of variance with Bonferroni post-hoc test was run to compare the match running demands and technical performance considering the ranking position. Shapiro–Wilk was used to test the normality of the sample and Levene's test for equality of error variance. Regarding the second aim of the study, Pearson’s correlation coefficient (*r*) was obtained to analyze the correlation between the total of points obtained at the end of the season and the variables related to match technical and running performance. The effect sizes were also reported as partial eta-squared (ηp^2^). The statistical analysis was performed on SPSS Statistics version 25 (IBM Corp., Armonk, NY, USA).

## Results

Table 1 shows the match technical performance required by teams in different ranking positions. In this regard, significant pairwise differences were observed based on the team ranking position since it had a significant interaction with the goals scored (high-ranked > medium-ranked > low-ranked teams), goals conceded (low-ranked > medium-ranked > high-ranked teams), shots on goal (high-ranked > medium-ranked and low-ranked teams), shots on goal allowed (low-ranked and medium-ranked > high-ranked teams), missed shots on goal (high-ranked > low-ranked teams), blocked shots on goal by any player (high-ranked > medium-ranked), saved shots on goal by the goalkeeper (high-ranked > medium-ranked and low-ranked teams), successful passes from open play (high-ranked > medium-ranked and low-ranked teams), unsuccessful passes from set pieces (low-ranked and medium-ranked > high-ranked teams), corner kicks (high-ranked > medium-ranked), assists (high-ranked > medium-ranked and low-ranked teams), aerial duels won (medium-ranked > high-ranked teams), aerial duels lost (low-ranked and medium-ranked > high-ranked teams), yellow–red cards (low-ranked > high-ranked teams), and percentage of ball possession (high-ranked > medium-ranked and low-ranked teams).

**Table 1:** Match technical performance by ranking position

Variables	High-ranked (Mean ± SD)	Medium-ranked (Mean ± SD)	Low-ranked (Mean ± SD)	F	*p*	ηp^2^
Goals scored	1.99 ± 0.38^b,c^	1.36 ± 0.28^a,c^	0.93 ± 0.11^a,b^	28.91	≤ 0.01	0.64
Goals conceded	1.24 ± 0.26^b.c^	1.54 ± 0.25^a.c^	1.86 ± 0.30^a.b^	12.09	≤ 0.01	0.42
Shots on goal	14.63 ± 1.93^b,c^	12.67 ± 1.74^a^	11.29 ± 0.70^a^	8.91	≤ 0.01	0.35
Shots on goal allowed	10.83 ± 1.72^b,c^	13.93 ± 1.01^a^	15.09 ± 1.33^a^	27.55	≤ 0.01	0.63
Missed shots on goal	12.69 ± 1.66^c^	11.33 ± 1.58	10.39 ± 0.68^a^	5.33	≤ 0.01	0.24
Shots off target	4.60 ± 0.82	4.19 ± 0.64	3.94 ± 0.32	2.35	0.11	0.13
Blocked shots on goal	3.54 ± 0.44^b^	3.07 ± 0.50^a^	3.11 ± 0.30	4.24	0.02	0.20
Saved shots on goal by GK	3.66 ± 0.54^b,c^	3.21 ± 0.47^a^	2.67 ± 0.25^a^	9.20	≤ 0.01	0.36
Successful passes from OP	417.13 ± 91.13^b,c^	297.46 ± 40.35^a^	286.66 ± 24.31^a^	16.28	≤ 0.01	0.50
Unsuccessful passes from OP	69.99 ± 5.75	69.94 ± 5.91	69.12 ± 2.29	0.06	0.94	0.00
Successful crosses from OP	2.52 ± 0.68^b,c^	2.43 ± 0.53^a^	2.13 ± 0.59^a^	0.87	0.43	0.05
Unsuccessful crosses from OP	7.91 ± 1.77	7.51 ± 1.61	7.74 ± 1.20	0.23	0.79	0.01
Successful passes from SP	18.59 ± 1.40	17.83 ± 1.33	18.94 ± 0.69	2.25	0.12	0.12
Unsuccessful passes from SP	4.80 ± 1.51^b,c^	6.76 ± 1.56^a^	7.12 ± 0.67^a^	8.20	≤ 0.01	0.33
Successful crosses from SP	1.78 ± 0.45	1.60 ± 0.30	1.41 ± 0.25	2.34	0.11	0.12
Unsuccessful crosses from SP	3.68 ± 0.63^b^	3.15 ± 0.38^a^	3.64 ± 0.42	5.06	≤ 0.01	0.24
Clearances	7.74 ± 2.46	9.73 ± 2.12	10.73 ± 3.48	3.62	0.04	0.18
Corner kicks	5.45 ± 1.12^b^	4.54 ± 0.52^a^	4.75 ± 0.75	4.76	0.02	0.22
Assists	1.40 ± 0.33^b,c^	0.90 ± 0.22^a^	0.62 ± 0.04^a^	23.21	≤ 0.01	0.58
Aerial duels won	20.16 ± 3.21^b^	23.88 ± 3.30^a^	23.25 ± 2.54	5.14	≤ 0.01	0.24
Aerial duels lost	19.54 ± 2.18^b,c^	23.85 ± 3.05^a^	24.57 ± 1.90^a^	11.83	≤ 0.01	0.42
Ground duels won	81.37 ± 4.90	77.10 ± 4.32	78.00 ± 4.43	3.27	0.05	0.17
Ground duels lost	78.59 ± 6.89	78.56 ± 4.06	79.17 ± 6.06	0.03	0.97	0.00
Counter attacks	0.24 ± 0.08	0.19 ± 0.12	0.12 ± 0.04	3.05	0.06	0.16
Offsides	2.19 ± 0.32	1.95 ± 0.46	1.84 ± 0.26	2.02	0.15	0.11
Fouls	11.27 ± 2.37^b^	12.92 ± 1.21^a^	12.15 ± 1.49	3.28	0.05	0.16
Fouls from the opponent	12.37 ± 1.51	11.93 ± 1.34	12.96 ± 1.37	1.27	0.29	0.07
Hand games	0.59 ± 0.15	0.62 ± 0.18	0.63 ± 0.14	0.21	0.81	0.01
Yellow cards	1.49 ± 0.33	1.72 ± 0.22	1.75 ± 0.14	3.72	0.04	0.18
Yellow–red cards	0.02 ± 0.03^c^	0.04 ± 0.04	0.07 ± 0.04^a^	4.54	0.02	0.22
Red cards	0.03 ± 0.04	0.03 ± 0.03	0.04 ± 0.04	0.39	0.68	0.02
Ball possession (%)	54.85 ± 4.79^b,c^	47.67 ± 2.23^a^	47.30 ± 1.64^a^	19.97	≤ 0.01	0.55

*SD* standard deviation, *OP* open play, *SP* set pieces, *GK* goalkeeper; ^a^statistical difference (*p* < 0.05) to high-ranked teams; ^b^statistical difference (*p* < 0.05) to medium-ranked teams; ^c^statistical difference (*p* < 0.05) to low-ranked teams

When it comes to match running performance (Table 2), there was a significant interaction between the ranking position and maximum speed (high-ranked > low-ranked teams), TD with ball possession (high-ranked > medium-ranked and low-ranked teams), SPD with ball possession (high-ranked > medium-ranked and low-ranked teams), SPA with ball possession (high-ranked > medium-ranked and low-ranked teams), and the TD without ball possession (medium-ranked and low-ranked > high-ranked teams).

**Table 2:** Match running performance by ranking position

Variables	High-ranked (Mean ± SD)	Medium-ranked (Mean ± SD)	Low-ranked (Mean ± SD)	F	*p*	ηp^2^
Maximum speed (km/h)	33.55 ± 0.43^c^	33.35 ± 0.27	33.05 ± 0.41^a^	3.97	0.03	0.19
TD with ball possession (km)	47.24 ± 6.92^b,c^	40.75 ± 9.15^a^	40.23 ± 4.05^a^	17.10	≤ 0.01	0.51
TD without possession (km)	42.79 ± 3.91^b,c^	47.80 ± 2.00^a^	47.57 ± 1.95^a^	12.71	≤ 0.01	0.44
SPD with ball possession (km)	2168.50 ± 164.02^b,c^	1925.48 ± 226.52^a^	1868.65 ± 68.47^a^	7.53	≤ 0.01	0.31
SPD without possession (km)	2107.46 ± 242.15	2129.92 ± 237.83	2141.17 ± 112.54	0.06	0.95	0.00
SPA with ball possession	105.52 ± 0.16^b,c^	93.41 ± 0.23^a^	91.84 ± 0.07^a^	10.25	≤ 0.01	0.38
SPA without possession	111.69 ± 11.15	113.81 ± 11.42	111.76 ± 5.50	0.17	0.84	0.01

*TD * total distance, *SPD* sprinting distance, *SPA* sprinting actions; ^a^statistical difference (*p* < 0.05) to high-ranked teams; ^b^statistical difference (*p* < 0.05) to medium-ranked teams; ^c^statistical difference (*p* < 0.05) to low-ranked teams

Regarding the second aim of the study for the correlation between match technical and running performance variables and teams’ success (Table 3), significant correlations were observed between the total of points obtained at the end of the season and the following technical performance variables: goals scored (*r* = 0.90; *p* < 0.001), assists (*r* = 0.88; *p* < 0.001), shots on goal allowed (*r* = −0.87; *p* < 0.001), goals conceded (*r* = −0.81; *p* < 0.001), ball possession (*r* = 0.78; *p* < 0.001), successful passes from open play (r = 0.77; *p* < 0.001), saved shots on goal by the goalkeeper (*r* = 0.70; *p* < 0.001), shots on goal (*r* = 0.64; *p* < 0.001), aerial duels lost (*r* = −0.63; *p* < 0.001), unsuccessful passes from open play (*r* = −0.63; *p* < 0.001), corner kicks (*r* = 0.56; *p* < 0.001), missed shots on goal (*r* = 0.53; *p* < 0.001), successful crosses from set pieces (*r* = 0.50; *p* = 0.002), clearances fouls (*r* = −0.48; *p* = 0.003), counter attacks (*r* = 0.44; *p* = 0.01), yellow cards fouls (*r* = −0.43; *p* = 0.01), shots off target (*r* = 0.40; *p* = 0.02), aerial duels won fouls (*r* = −0.38; *p* = 0.02), unsuccessful crosses from set pieces (*r* = 0.35; *p* = 0.04), blocked shots on goal (*r* = 0.35; *p* = 0.04), successful crosses from open play (*r* = 0.35; *p* = 0.04), fouls (*r* = −0.34; *p* = 0.04), and ground duels won (*r* = 0.33; *p* = 0.04).

**Table 3:** Pearson correlation coefficients (r) between the total of points obtained at the end of the season and match technical and running performance variables

Variables	*r*	*p*
Goals scored	0.90	< 0.001
Assists	0.88	< 0.001
Ball possession (%)	0.78	< 0.001
Successful passes from OP	0.77	< 0.001
TD with ball possession (km)	0.75	< 0.001
Saved shots on goal by GK	0.70	< 0.001
Shots on goal	0.64	< 0.001
Corner kicks	0.56	< 0.001
SPA with ball possession	0.55	< 0.001
Missed shots on goal	0.53	< 0.001
Successful crosses from SP	0.50	0.002
SPD with ball possession (km)	0.49	0.002
Counter attacks	0.44	0.01
Maximum speed (km/h)	0.41	0.01
Shots off target	0.40	0.02
Unsuccessful crosses from SP	0.35	0.04
Blocked shots on goal	0.35	0.04
Successful crosses from OP	0.35	0.04
Ground duels won	0.33	0.04
Unsuccessful crosses from OP	0.29	0.09
Offsides	0.27	0.11
Unsuccessful passes from OP	0.06	0.73
Successful passes from SP	−0.07	0.67
SPA without possession	−0.11	0.51
Hand games	−0.12	0.48
SPD without possession (km)	−0.12	0.48
Ground duels lost	−0.15	0.39
Red cards	−0.16	0.36
Fouls from the opponent	−0.16	0.34
Yellow–red cards	−0.30	0.07
Fouls	−0.34	0.04
Aerial duels won	−0.38	0.02
Yellow cards	−0.43	0.01
Clearances	−0.48	0.003
Unsuccessful passes from SP	−0.63	< 0.001
Aerial duels lost	−0.63	< 0.001
TD without possession (km)	−0.70	< 0.001
Goals conceded	−0.81	< 0.001
Shots on goal allowed	−0.87	< 0.001

*OP* open play; *SP* set pieces; *GK* goalkeeper; *TD* total distance; *SPD* sprinting distance; *SPA* sprinting actions

In addition, significant correlations were observed between the total of points obtained at the end of the season and the match running performance variables such as TD with ball possession (*r* = 0.75; *p* < 0.001), TD without ball possession (*r* = -0.70; *p* < 0.001), SPA with ball possession (*r* = 0.55; *p* < 0.001), SPD with ball possession (*r* = 0.49; *p* = 0.002), and maximum speed (*r* = 0.41; *p* = 0.01).

## Discussion

The aims of this study were to determine the match technical and running performance required by different teams and analyze the correlation between teams’ success at the end of the season and variables related to match technical and running performance. The main findings were that there was a significant interaction between the ranking position and both match technical and running performance. Also, significant correlations were observed between the total of points obtained at the end of the season and the match technical and running performance. However, goals scored, assists, allowed shots on goal, goals conceded, ball possession ratio, successful passes from open play, total distance covered (with and without ball possession), and saved shots on goal by the goalkeeper were the variables with the strongest correlation (*r* > 0.7; *p* ≤ 0.01) with the total of points obtained at the end of the season. In addition, another main finding of this study was the importance of running performance at high intensity given the moderate correlations observed between team success and both SPD and SPA with ball possession (*r* = 0.49–0.55; *p* < 0.002).

Our analysis indicated that significant interactions were observed between the ranking position and both physical and technical performance, which is in line with previous studies [5, 7–9, 13, 23]. From a technical performance perspective, higher-ranked teams had better performance than lower-ranked teams in variables related to offense (e.g., ball possession, corners, shots on goal, or successful passes) [7, 8] and defense (e.g., fouls or yellow cards) [23]. In addition, the total amount of saved shots on goal by the goalkeeper were key to determine the ranking position, which emphasizes the importance of this position in modern soccer [24]. However, match running performance had a significant interaction with the ranking position as well. A previous study concluded that more successful teams may exert less physical effort in match play probably because of a greater technical ability or tactical awareness, which lead to greater ball possession [25]. In this regard, our study confirmed that higher-ranked teams covered significantly greater TD with ball possession than lower-ranked teams while lower-ranked teams covered significantly greater TD without ball possession [9, 13, 18]. Also, it is to mention that the sprinting actions with ball possession performed by high-ranked teams were greater compared to lower-ranked teams, which implies that sprinting actions are key for tactical purposes. Specifically, these actions allow to generate open spaces, 1 on 1 events, or penetrative passes, which reinforces the importance of strength and conditioning in professional soccer [9, 26–28].

Furthermore, previous research has shown that technical performance may be more important than physical performance for success in soccer [3, 29]. Our study clearly confirmed this hypothesis considering that goals scored (*r* = 0.90; *p* ≤ 0.01), saved shots on goal by the goalkeeper (*r* = 0.70; *p* ≤ 0.01), assists (*r* = 0.88; *p* ≤ 0.01), allowed shots on goal (*r* = −0.87; *p* ≤ 0.01), goals conceded (*r* = −0.81; *p* ≤ 0.01), ball possession ratio (*r* = 0.78; *p* ≤ 0.01), and successful passes from open play (*r* = 0.78; *p* ≤ 0.01) were the variables with the greatest correlation with the total of points obtained at the end of the season in the German Bundesliga. This is in line with a recent study investigation in the same league, which showed that accuracy-related variables (e.g., shots on target or goal efficiency) are critical for team success [5, 10]. In consequence, teams should concentrate on developing team strategies to create more frequent situations to shoot on goal. For instance, one way is to speed up the game by reducing the number of foot contacts with the ball even though appropriate technical training is needed as well. Nevertheless, there were other match technical and running performance variables with high correlation with the total of points obtained at the end of the season [9, 13, 18]. For instance, teams keeping the ball, which means that they run more distance with the ball, perform more successful passes/crosses, and avoid clearances, are more successful at the end of the season. Thus, training drills with special focus on these technical and physical actions need to be designed.

The authors are fully aware of the many factors that might have influenced the results of the analyses presented here. For example, a limitation of this study are different speed thresholds to calculate sprinting actions might be selected (e.g., 24 or 25 km/h) and variables such as high-speed running distance (e.g., above 19.8 km/h) and total of accelerations or decelerations might be included in the analysis if these are reported by the tracking system [30, 31]. Another limitation is that research should be developed considering the influence of different contextual variables (e.g., playing position or fitness) [32]. In addition, the present study was focused on a specific league (i.e., Bundesliga). Also, no internal load data (e.g., heart rate or rate of perceived exertion) were collected. Therefore, future investigations may consider these limitations to have a better understanding of both physical and technical performance in professional soccer. Moreover, running performance on the sample of the whole game should be analyzed to have a better understanding of how running parameters influence the game outcome.

## Conclusions

Strength and conditioning coaches may consider the results from this study to develop adequate training strategies since significant interactions were observed between the ranking position and both physical and technical performance. Teams should concentrate on developing strategies to create more frequent situations to shoot on goal. For instance, training drills measures may be adapted to medium and large games, in which teams may compete according to the principles of irrelevance and numerical superiority. Games such as 8 vs 6, 9 vs 7, or 10 vs 8 might be a natural environment that elicit many situations ending with a goal. Also, games including neutral players, who may support the team having the ball in the attacking phase, can contribute to creating goal situations. In addition, it is required that teams learn to keep the ball, which implies running more distance with the ball, performing more successful passes/crosses, and avoiding clearances, in order to be more successful at the end of the season. Thus, training drills with special focus on these technical and physical actions need to be designed. Also, considering that sprinting actions with ball possession performed by high-ranked teams were greater compared to lower-ranked teams, this implies that that the development of sprint performance in match play is necessary. For example, this might be trained including transition games. Finally, given the correlation between the total of points obtained at the end of the season and the total of saved shots on goal by the goalkeeper in this league, coaches and scouts should consider the importance of this position for team success. Hence, these observations may have practical implications showing the direction in which professional soccer teams (weaker teams, especially) may develop the game.

## Data Availability

Restrictions apply to the availability of the data analyzed during the current study considering that it is based on data collected in the context of professional soccer matches to allow all clubs of the league to match data analysis, and no informed consent was required by the Ethics Committee.

## Abbreviations

TD: Total running distance
SPD: Sprinting distance
SPA: Sprinting actions
ηp^2^: Partial eta-squared
